# A Pediatric Case of Diffuse Alveolar Hemorrhage Secondary to Poststreptococcal Glomerulonephritis

**DOI:** 10.1155/2017/1050284

**Published:** 2017-12-20

**Authors:** Alison Markland, Gregory Hansen, Anke Banks, Rajni Chibbar, Darryl Adamko

**Affiliations:** ^1^University of Saskatchewan, Saskatoon, SK, Canada; ^2^Division of Pediatric Intensive Care, University of Saskatchewan, Saskatoon, SK, Canada; ^3^Department of Pediatrics, Cumming School of Medicine, University of Calgary, Calgary, AB, Canada; ^4^Department of Laboratory Medicine, University of Saskatchewan, Saskatoon, SK, Canada; ^5^Division of Pediatric Respirology, University of Saskatchewan, Saskatoon, SK, Canada

## Abstract

This report summarizes a case of a 4-year-old girl with poststreptococcal glomerulonephritis and diffuse alveolar hemorrhage, an atypical presentation in this age group and type of vasculitic disease. We propose that her rapid improvement in clinical status was due to her treatment, continuous renal replacement therapy (CRRT). This mechanism would have impacted recovery by removing factors such as endothelial microparticles, superantigens, and immune complexes that have been postulated as the pulmonary-renal link. This may be an interesting avenue of exploration going forward given the lack of evidence in treating such conditions and emergence of CRRT.

## 1. Introduction

Diffuse alveolar hemorrhage (DAH) can be a life-threatening complication following numerous causes including respiratory infection, vasculitic diseases, or malignancy. Of the vasculitides in children, DAH is most commonly associated with granulomatosis and polyangiitis or antiglomerular basement membrane (anti-GBM) vasculitis. However, association of poststreptococcal glomerulonephritis (PSGN) with DAH is extremely rare; of the five cases reported only one was pediatric. In this report, we discuss a second case of a young child presenting with severe respiratory distress and significant hemoptysis who dramatically improved with continuous renal replacement therapy (CRRT).

## 2. Case

A 4-year-old female presented to the Pediatric Emergency Department at Royal University Hospital in Saskatoon, SK, Canada, with 4 days of worsening cough and increased work of breathing and one day of anuria. Her initial vital signs showed a temperature of 37.5 degrees Celsius, pulse rate of 125 beats per minutes, blood pressure of 114/58 mmHg, respiratory rate of 70 breaths per minute, and oxygen saturation of 95% on 30 litres of high flow oxygen with 21% FiO2. On exam, she had bilateral periorbital edema, crusted nasal discharge, and pallor. Rash and purpura were absent. She had nasal flaring, intercostal retractions, and coarse crackles bilaterally although more prominent on the right.

She had cough, sore throat, and rhinorrhea at a walk-in clinic approximately one-week prior and was given a prescription for amoxicillin. She also had iron deficiency anemia six months prior and started oral iron supplementation. She otherwise had no significant past medical history, significant travel history, or recent infectious contacts. Her mother, however, did have a history of treated tuberculosis (TB). Her immunizations were up to date.

Initial laboratory investigations revealed white blood cell count 9.08 × 10^9^/L, decreased hemoglobin 60 g/L, and normal platelet count of 349 × 10^9^/L. Her urea (16.9 mmol/L) and creatinine (46 umol/L) were elevated. She had a slightly elevated CRP (34.5 mg/L), normal glucose (6.7 mmol/L), normal sodium (142 mmol/L), high potassium (5.9 mmol/L), mildly elevated chloride (112 mmol/L), and a low bicarbonate (15 mmol/L). Her D-dimer was 929 ug/L, APTT was low-normal (22 seconds), and fibrinogen was normal (3.42 g/L). Her urinalysis demonstrated leukocyte esterase 500 WBC/uL, protein 1.5 g/L, and blood 250 RBC/uL, with negative nitrites. Urine microscopy revealed leukocytes 20–50 WBC/HPF, erythrocytes 11–20 RBC/HPF, and granular casts 3–5/LPF.

**Figure 1:**
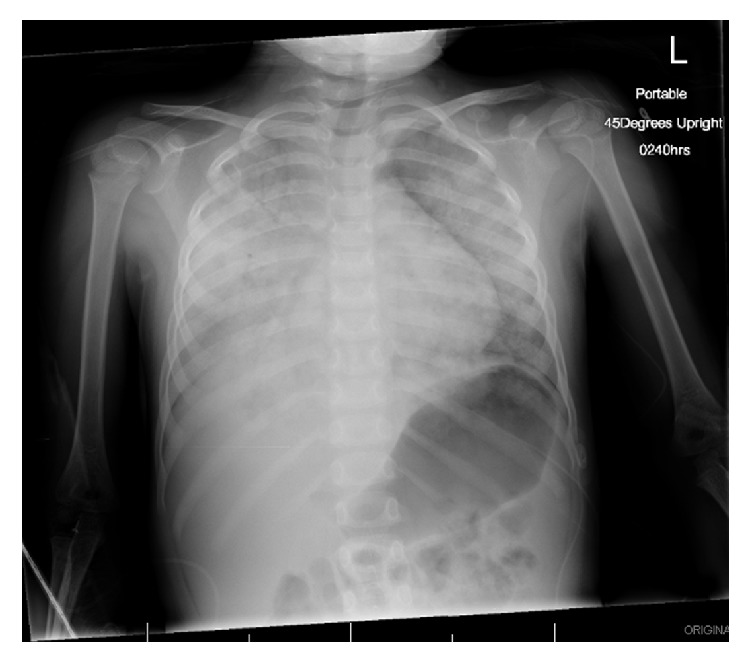
Radiograph on day of admission. Radiograph showing dense consolidation of the right lung obscuring the cardiothymic silhouette and tracheal deviation towards the right. Bilateral consolidation with associated air bronchograms.

She was transferred to the Pediatric Intensive Care Unit (PICU) after over 250 mL of hemoptysis. Her initial arterial gas showed a normal anion gap metabolic acidosis with concomitant respiratory acidosis (pH 7.25, carbon dioxide 43 mmHg, bicarbonate 18 mmol/L, and corrected anion gap 13.5). Her first chest X-ray showed bilateral consolidations with air bronchograms, consistent with diffuse pulmonary hemorrhage (Figure 1). She was initiated on bilevel positive airway pressure, but she continued to deteriorate with worsening respiratory acidosis (arterial pH 7.01, carbon dioxide 73 mmHg, and bicarbonate 18 mmol/L). She was intubated and shortly thereafter required high frequency oscillation ventilation (HFOV).

With our suspected diffuse alveolar hemorrhage, an upper gastrointestinal bleed (GI) was considered in the differential diagnosis. We elected not to pursue investigations of the GI tract, because her initial management in the PICU after intubation was revealing. A nasogastric tube was promptly inserted which did not indicate any evidence of gastric blood. More importantly, initial tracheal aspirates from closed inline suctioning revealed bright red blood, with subsequent aspirates suggesting a mixture of bright red and congealed blood. With her microcuffed endotracheal tube inflated and routinely monitored (q12 hrs) for minimum inflation pressures, risks for significant aspiration should have been mitigated.

She received two transfusions of packed red blood cells to correct the anemia. Given her worsening clinical status and anuria, CRRT was initiated. This led to a rapid improvement in her electrolyte abnormalities. Further investigations revealed positive perinuclear anti-neutrophil cytoplasmic antibody (p-ANCA) with anti-myeloperoxidase antibody IgG 30, and negative cytoplasmic ANCA (c-ANCA) anti-proteinase 3 antibody IgG 2. Both complements C3 (0.18 g/L) and C4 (0.10 g/L) were low. Anti-glomerular basement membrane (GBM), anti-phospholipid, and antinuclear antibodies were negative.

Although vasculitis was strongly suspected due to the constellation of pulmonary and renal findings, infectious causes were also considered. Nasopharyngeal swab for rhinovirus was positive. Blood, urine, and lower respiratory cultures were negative.* Mycobacterium tuberculosis* polymerase chain reaction (PCR), acid fast bacilli stain,* Bordetella*,* Mycoplasma pneumoniae*, and* Hantavirus* PCR were also negative.

**Figure 2:**
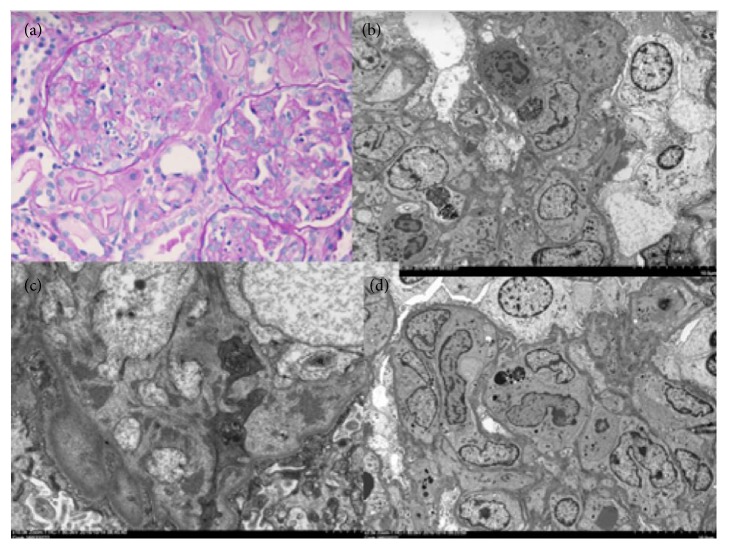
Renal biopsy. (a) Acute diffuse proliferative glomerulonephritis with intracapillary neutrophils and closure of glomerular capillaries and infiltration of capillaries. (b) and (c) Portion of glomerus shows intracapillary inflammatory cells, swelling of endothelial cells, mesangium, and subendothelial and (d) rare subepithelial dense deposits.

A renal biopsy was performed which demonstrated enlarged glomeruli with diffuse endocapillary hypercellularity with numerous neutrophils and closure of glomerular capillaries (Figure 2). There was endothelial cell swelling but no areas of glomerular capillary wall necrosis or cellular crescents. On immunofluorescent histology, C3 stain showed glomeruli with finely granular 3+, irregular diffuse staining of capillary walls, and mesangium (Starry sky pattern) and IgG demonstrated 1-2+ focal segmental, granular capillary wall staining. There was no immunopositivity with IgA antibody. Electron microscopy confirmed the increased numbers of endocapillary and infiltrative inflammatory cells in the glomerular tuft as well as swelling of endothelial cells. Scattered mesangial, subendothelial deposits were present. However, the classical subepithelial “hump like” deposits were rare. The case was also reviewed by pediatric nephropathologist to confirm the diagnosis of PIGN and to rule out C3 nephropathy. Following the biopsy, a streptozyme test was done which was positive with a titre of 1 : 100.

**Figure 3:**
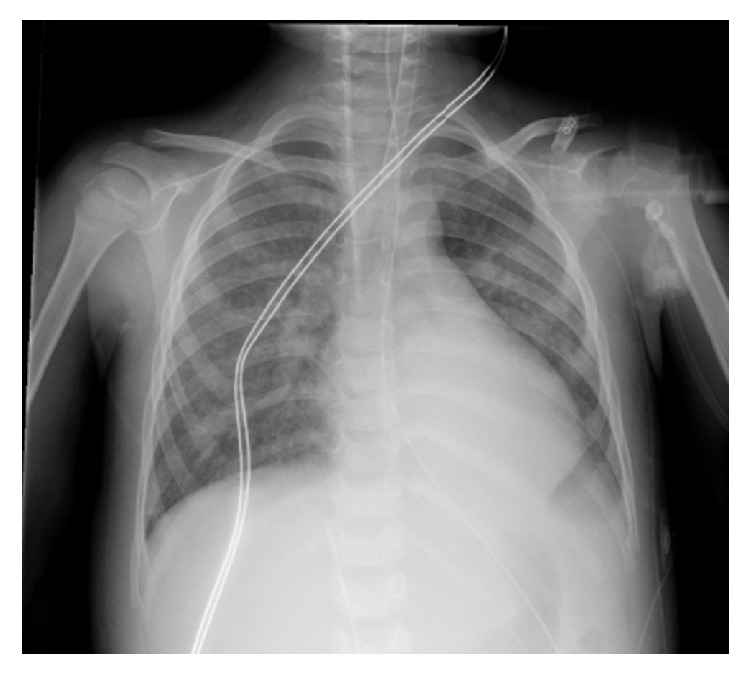
Follow-up radiograph. Improvement in pulmonary disease from previous image five days following admission and treatment. Mild perihilar consolidation persists with basal atelectasis.

High dose methylprednisolone therapy was initiated under the suspicion of vasculitis following renal biopsy. Upon receiving the results of the biopsy 3 days after admission, corticosteroid was stopped and emphasis was given to supportive treatment. Her chest X-ray improved greatly by day 5 in hospital (Figure 3) and she was successfully weaned off HFOV and CRRT. She subsequently developed systemic hypertension, which was managed with captopril and amlodipine. At the age of 4 years she was too young for pulmonary function tests at our institution, and a CT chest did not seem warranted given her clinical improvement.

She was evaluated in pulmonary and nephrology follow-up clinics 3 months after discharge. There was no further history of cough, shortness of breath, or anemia and the CXR completely cleared. Her C3 and C4 normalized and her blood pressure remained normotensive. She will receive long-term monitoring for respiratory and renal impairment, but she is expected to have a complete recovery.

## 3. Discussion

Herein, we present a case of a child suffering DAH in the setting of PSGN. PSGN leading to pulmonary disease has been described in only a few cases and is exceedingly rare in the pediatric population [1–7]. This case highlights a unique presentation of PSGN with DAH, signs of fluid overload, AKI, severe respiratory compromise that quickly resolved with a short course of systemic corticosteroids, and CRRT.

Poststreptococcal glomerulonephritis is globally the most common cause of acute nephritis in children [8]. Following streptococcal infection of the respiratory tract or skin with a nephritogenic strain of Group A beta hemolytic* Streptococcus*, kidney injury may occur. The putative mechanism favored for glomerulonephritis following streptococcal infection is immune complex formation deposition in the glomerulus. However, the exact pathophysiology is largely unknown with proposed mechanisms including trapping of circulating immune complex versus in situ immune complex formation [9–11]. Diagnosis of PSGN is made with evidence of streptococcal infection either by documented culture or antibody titers in the presence of acute nephritis. Although renal biopsy is not routinely part of the diagnostic formulation in PSGN, it may be useful if there is no documented evidence of streptococcal infection or in cases of recurrence [12]. On histological exam, focal segmental proliferative or mesangioproliferative patterns are the typical types of glomerular injury documented. C3 dominant staining with subepithelial “humps” and subendothelial deposits are commonly seen [13].

The common link between lung and kidney disease is likely related to similar cellular morphology of the vessels with a similar response to immune disorders. In the case of immune mediated hemorrhage in the lung, the hypotheses for vessel leak are (1) vessel injury from neutrophil activation (e.g., through antineutrophil antibodies (ANCA associated vasculitis, AAV)), (2) immune complex deposition with activation of complement, anaphylatoxin, and mast cells, or (3) abnormal lymphocyte inflammation and injury [14–16]. For example, infection with* Staph. aureus* or* Streptococcus* species is associated with exacerbation of AAV kidney disease. These organisms can act as superantigens, which activate T cells to injury vessels. High circulating endothelial microparticles (EMP) have been identified in children with other nephritic syndromes such as Henoch-Schönlein purpura [17]. High EMP concentrations may also induce significant lung pathology, including pulmonary edema, neutrophil recruitment, and endothelial-alveolar barrier compromise [18]. The mechanism of pulmonary hemorrhage in our case is unknown but we suggest that her biopsy would be in keeping with a form of AAV, though the role of superantigen is another factor to consider.

The patient in the previously reported that pediatric case of PSGN related DAH also required treatment in the intensive care unit and was similarly treated with corticosteroids for a brief period [2]. However, she was not ill enough to require CRRT or HFOV. It is difficult to say whether corticosteroids had an impact on resolution of symptoms and prognosis, as supportive therapy alone is the mainstay of treatment, with no evidence that other therapies offer a therapeutic advantage [19]. It is possible that the steroids were not necessary and this was the natural course of disease. In contrast, one case report by De Torrente et al. indicated that corticosteroids were necessary to treat pulmonary hemorrhage with poststreptococcal glomerulonephritis in an adult [20].

While it is plausible that the steroid pulse may have altered her clinical trajectory, we also propose the potential positive therapeutic effect of continuous renal replacement therapy (CRRT) on diffuse alveolar hemorrhage. Recently, a CRRT model utilizing a filter pore size of 200 *μ*m significantly reduced EMP concentrations over a short duration [21]. We used a 69 CRRT hemofilter (Baxter Gambro). Removal of factors like EMP, anti-neutrophil antibodies, immune complexes, or superantigens could have been the important factor expediting recovery. Future directions of research may involve investigation of the use of CRRT in pulmonary-renal syndrome.

Prognosis for pediatric patients following PSGN is good. Only 10% of pediatric patients affected by PSGN will go on to have persistent hematuria or proteinuria; fewer still will have chronic kidney disease [22]. Prognosis for children with DAH is less predictable. Pulmonary hemorrhage from anti-GBM vasculitis has led to long-term deficits in pulmonary function, but recurrence of DAH has been reported. Patients with DAH secondary to idiopathic pulmonary hemosiderosis, however, are reported to have long standing deficits in lung function and higher mortality [23].

## 4. Conclusion

Presented above is a case of a 4-year-old in significant distress due to DAH resulting from PSGN. This presentation is rare and has been described in only one other instance in a child. This atypical presentation was followed by a rapid improvement in clinical presentation and symptoms following CRRT. We propose that CRRT impacted recovery by removing factors such as EMP, superantigens, and immune complexes that have been postulated as the pulmonary-renal link. This may be an interesting avenue of exploration going forward given the lack of evidence in treating such conditions.
